# Von Hippel-Lindau Disease and the Eye

**DOI:** 10.18502/jovr.v15i1.5950

**Published:** 2020-02-02

**Authors:** Saeed Karimi, Amir Arabi, Toktam Shahraki, Sare Safi

**Affiliations:** ^1^Ophthalmic Research Center, Shahid Beheshti University of Medical Sciences, Tehran, Iran; ^2^Department of Ophthalmology, Torfeh Medical Center, Shahid Beheshti University of Medical Sciences, Tehran, Iran; ^3^Ophthalmic Epidemiology Research Center, Shahid Beheshti University of Medical Sciences, Tehran, Iran

**Keywords:** Diagnosis, Retinal Capillary Hemangioma, Treatment, Von Hippel-Lindau

## Abstract

Retinal hemangioblastoma (also referred to as retinal capillary hemangioma) is a benign lesion originating from the endothelial and glial components of the neurosensory retina and optic nerve head. Historically known as a manifestation of the von Hippel-Lindau (VHL) disease, it can be seen as an isolated finding or in association with some rare ocular conditions. In addition to characteristic ophthalmoscopic features, results of numerous ancillary tests including angiography, ultrasound, optical coherence tomography, and genetic tests may support the diagnosis and differentiate it from similar conditions. Because of serious life-threatening complications of VHL disease, every ocular approach to retinal hemangioblastomas should be in relationship with additional multidisciplinary diagnostic and therapeutic efforts. In addition, any patient with actual or probable diagnosis of VHL disease should be screened for ocular involvement. Unfavorable visual loss can occur early, and ocular complications of VHL range from exudative retinopathy to tractional retinal detachment, neovascular glaucoma, and phthisis bulbi. Accordingly, various treatment methods have been tested with overall acceptable responses, including photocoagulation, cryotherapy, photodynamic therapy, plaque radiotherapy, vitrectomy, and more novel intravitreal injections of anti-vascular endothelial growth factors and propranolol.

##  INTRODUCTION 

Retinal hemangioblastoma (RH), also known as retinal capillary hemangioblastoma, is a benign vascular neoplastic lesion originating in the neurosensory retina or optic disc. Vigla described

hemangioblastoma in a patient who died of central nervous system (CNS) lesions for the first time in 1864.^[1]^ Hemangioblastoma presents as a highly vascular, and well-bordered, slowly growing neoplastic lesion that contains a mixture of stromal cells, endothelial cells, pericytes, and mast cells.^[2]^ RHs are usually observed in von Hippel–Lindau (VHL) disease, which is an autosomal dominantly inherited condition, in which mutations in the VHL tumor suppressor gene is believed to cause the development of characteristic benign and malignant, mainly vascular, neoplasms or cysts in the CNS and internal organs.

RH is one of the earliest and most frequent manifestations of VHL disease.^[3]^ Sporadic RH can also occur in the absence of VHL disease. According to the Webster et al's report, the features of sporadic retinal hemangiomas including age of presentation, degree of visual morbidity, complications, morphology, and anatomic location of tumors are indistinguishable from those seen in the VHL disease.^[4]^

The prevalence of VHL was reported as 30–58% among patients with RHs.^[5]^ In the study by Niemelä et al including 36 patients with retinal hemangioblastomas, 11 cases had definite clinical diagnosis of VHL and ten cases were diagnosed with clinically suspected VHL.^[5]^ In the same study, visual prognosis of affected individuals was more favorable in non-VHL patients than in VHL patients, which was in contrast with the finding of the study mentioned earlier.^[4]^ Some reports have disclosed that RHs exist in association with other retinal conditions, such as chorioretinal coloboma and Marshall–Stickler syndrome.^[6,7]^

Given the life-threatening nature of some of the complications and manifestations of VHL, timely intervention needs appropriate surveillance, and proper diagnosis can be made based on clinical criteria and genetic evaluation for mutations in the VHL gene.

##  METHOD

We searched PubMed and the Web of Knowledge databases to extract all published studies about VHL disease from inception to March 2019. “Von Hippel-Lindau”, “Retinal capillary hemangioma”, “Diagnosis”, and “Treatment” were used as the keywords. We also reviewed the reference lists of related studies. No language restriction was applied. Two independent investigators screened the abstracts and titles of extracted articles to determine the eligible publications. They reviewed the full text of the pertinent articles. Discrepancies were resolved through consensus.

##  VHL Disease

VHL disease is an exceedingly penetrant, autosomal dominantly inherited, multisystem neoplasia disorder caused by mutations in the VHL gene. Although VHL is hereditary in the majority of cases, new mutations are the cause in up to 20% of the cases.^[8]^ Cardinal manifestations include brain and spinal cord hemangioblastoma, renal cell carcinoma (RCC), RH, pheochromocytoma, epididymal and broad ligament cystadenomas, endolymphatic sac tumor, pancreatic neuroendocrine tumors, and renal and pancreatic cysts.^[9]^ The approximate incidence of VHL disease is 1 in 36,000 live births, and the penetrance is over 90% by 65 years of age.^[10]^ The most reported causes of mortality are metastasizing RCC and CNS lesions,^[11]^ and despite advances in clinical management, life expectancy for VHL patients remains low at 40–52 years.^[8]^ However, improvements in early diagnosis, surveillance, and treatment have led to better prognoses, and an expert multispecialty team is necessary for the optimal management of this complex disease.

##  History

The first pieces of the VHL syndrome puzzle began to be recognized in the late 19th century.^[12]^ These earliest findings of the disease have been summarized by Melmon and Rosen.^[13]^ In 1904, von Hippel published clinical data describing the features of a retinal disease in two patients and seven years later, he had been equipped to perform a histological examination on a subsequently enucleated eye of one of his patients; von Hippel named the pathological findings “angiomatosis retinae.” In 1926, the Swedish pathologist Lindau revealed the relationship between retinal and cerebellar hemangioblastomas and their association with cysts in some viscera including the kidney, epididymis, and pancreas as components of a hereditary syndrome. Accordingly, a CNS hemangioblastoma was known as a “Lindau tumor”, while the identical retinal lesion came to be known as a “von Hippel tumor”. By the time Melmon and Rosen published their review, the name of the familial syndrome was “Lindau disease”. This term was changed to “von-Hippel Lindau (VHL)” disease in the 1970s. Melmon and Rosen published the initial clinical diagnostic criteria for VHL disease in a landmark paper in 1964.^[13]^ Seizinger and colleagues explained the linkage of the VHL gene to chromosome 3 in 1988,^[14]^ and shortly thereafter, in 1993, Latif and colleagues discovered the VHL tumor suppressor gene.^[15]^

##  Mechanism of Cellular Dysfunction

VHL disease is caused by some mutations in a tumor suppressor gene, the VHL gene, present on chromosome 3 (3p25-26).^[15]^ The product of the gene is the VHL protein (pVHL), which has been found to participate in cellular oxygen sensing. Understanding of mechanisms related to the VHL gene has provided insight into cell signaling and function under normoxic and hypoxic conditions.^[16]^ VHL gene contains three exons that produce two distinct spliced mRNAs. The mRNAs differ in the presence or absence of exon number 2, known as Isoform I and II, respectively. Although it has been believed that the second isoform does not produce any endogenous tumor suppressor protein and the Isoform I is the only form that results in functional protein, expression of the uncharacterized protein isoform pVHL172, which is translated from Isomer II, is shown to upregulate a subset of pro-tumorigenic genes including TGFB1, MMP1, and MMP13.^[17]^ Following ubiquitous expression of VHL, Isoform 1 encodes two isoforms of pVHL, which comprises 213 amino acids and 160 amino acids, respectively, and both of these isoforms have tumor suppressor activity.^[18]^ pVHL, which is a part of the ubiquitin ligase complex and serves as the substrate-recognition subunit, assigns proteins for proteasomal degradation. Hypoxia-inducible factor 1α (HIF-1α) and hypoxia-inducible factor-2α (HIF-2α) are among the targets of this ubiquitin ligase, which undergo prolyl-hydroxylation under normoxic conditions, authorizing for binding to pVHL and activation of ubiquitin peptides that result in proteasomal degradation of HIFs.^[19,20]^ It is clear that in the absence of normal pVHL, HIF-1α and HIF-2α are not degraded, but form heterodimers with hypoxia-inducible factor 1β (HIF-1β), and produce transcription factors for a wide array of over 800 genes.^[16]^ Upregulation of cell survival proteins by HIF signaling, such as epidermal growth factor receptor and transforming growth factor alpha, in addition to angiogenesis factors, for instance vascular endothelial growth factor (VEGF) and platelet-derived growth factor (PDGF), are hypothesized to play a key role in the development of neoplastic vascular lesions in VHL disease, including RH.^[21,22,23,24,25]^ The significance of other actions of pVHL independent of HIF signaling is unclear in VHL disease pathogenesis.^[16]^

VHL disease is mostly received from a mutant copy of the VHL gene from an affected parent and a normal copy from the other parent. Knudson's two-hit model for tumorigenesis indicated that the somatic inactivation of the normal allele in one or more cells, in combination with a germline mutation in the other allele, causes the mutation to clinically manifest.^[26]^ In a large study on 181 kindreds with VHL disease, 42 cases (23%) did not have any relevant family history, indicating a putative first-generation diagnosis.^[27]^ In these cases, mosaicism may explain the underlying germline transmission from the unaffected parent. Additionally, it can explain occasional cases in which VHL disease manifests clinically but initial genetic testing is negative for mutation.^[28]^ Stolle and colleagues published a report in 1998 on different genetic testing methods that helped in the identification of germline mutations in 100% of families with VHL disease, establishing the importance of VHL gene testing for the diagnosis of this condition.^[29]^

Mutations of VHL are highly varied, ranging from the base pair substitution in a single amino acid codon to the complete deletion of the gene.^[30,31]^ Many of these mutations target pVHL regulation of HIF signaling. However, the heterogeneous manifestations of VHL disease suggest that different mutations may affect pVHL-associated cellular mechanisms in different ways.^[32]^ The relationship between the type of VHL mutation and the severity and prevalence of ocular complications has been investigated in several studies with contradictory results.^[33]^ Initial findings denied any association between the type of VHL mutation and visual function.^[34]^ However, it is now believed that partial deletion, missense, and nonsense mutations are correlated with higher prevalence of RHs and worse visual prognosis, unlike complete deletion of the VHL gene.^[35,36]^ Additionally, the location of a missense mutation in VHL correlates with the phenotype of ocular VHL disease, and mutations in the alpha(a)-domain are associated with a higher rate of retinal and optic nerve hemangioblastomas than those in the beta(b)-domain.^[37]^ Different VHL gene mutations in four VHL families support the genotype–phenotype correlations.^[33]^

##  Diagnosis

Retinal and CNS hemangioblastomas are two main clinical features of VHL disease and the other systemic abnormalities have less diagnostic significance. However, variability in systemic abnormalities is an important feature of this disease. Only one systemic involvement manifests in some cases and not all abnormalities present together in many individuals. The diagnosis can be made when there are two hemangioblastomas, or one of them in combination with a visceral manifestation. It is notable that a positive family history is as valuable as a CNS tumor in the diagnosis; one index tumor, such as hemangioblastoma, RCC, or pheochromocytoma, in association with a positive family history is sufficient to diagnose VHL disease.^[38]^ Genetic testing is useful in challenging cases for screening of at-risk family members of a patient with VHL disease with no family history or visceral lesions.

##  Screening and Surveillance

Different guidelines have been published for screening of VHL disease. Among these guidelines, the ones designed by Choyke et al have suggested urinary catecholamine testing, ophthalmoscopy, brain magnetic resonance imaging, and abdominal computed tomography or ultrasound for early detection of the manifestations.^[39]^ In the screening guideline, ophthalmoscopy is recommended to begin from infancy and to be repeated yearly.

As VHL disease is complex, optimal management involves care by a multispecialty team with expertise in ophthalmology, otolaryngology, neurosurgery, endocrine surgical oncology, neuroradiology, urology, pathology, genetics, and rehabilitation medicine.

##  Ocular Manifestation of VHL Disease

Although RH is one of the most common clinical manifestations of VHL disease, the precise prevalence of ocular involvement is difficult to ascertain from case series. Singh et al reported that retinal capillary hemangioma is the most frequent and the earliest manifestation of VHL disease. The frequency of occurrence has been reported to vary from 49% to 85%.^[40]^ In another article, it is claimed that RHs are seen in as many as 60% of the patients, being the second most frequent manifestation after CNS hemangioblastomas.^[8]^ Whether the most frequent or not, RHs are often the first manifestation. Although retinal lesions are hamartomas in nature, they are usually not present at birth.^[41]^ The mean and median age of onset is 25 and 21, respectively, which is the lowest among the other clinical features.^[9,42]^ RH often manifests as a solitary lesion. However, around one-third of patients may have multiple retinal hemangiomas and up to half of the patients may present with bilateral involvement.^[40]^ It has been shown that there is no effect of gender on the laterality and severity of the ocular involvement.^[43]^

In a large cross-sectional study in which participants were identified based on a diagnosis of VHL disease independent of ophthalmologic features, 335 of 890 patients from 220 unrelated pedigrees were found to have ocular involvement.^[42]^ Of those with ocular disease, 42% had unilateral involvement and 58% had bilateral involvement. Affected patients were from 7 to 84 years old (mean – 36 years old), and 45% were male. In the study, RHs of VHL were found in all ages and racial groups, and in both sexes. Results of the study confirmed the findings of one previous study about the lack of effect of sex on the ocular phenotype of VHL patients. Among the main demographic features considered in the study, only age was found to have an effect on ocular phenotype, and only in some respects. However, the authors did not find a significant correlation between increasing age and either the laterality, number of RHs per eye, or the extent of peripheral retina angiomatosis. This suggests that the probability for the formation of new lesions in the eye may not remain constant over time. Webster et al^[43]^ also did not find an age correlation to number of tumors. Dollfus et al^[44]^ reported increases in RH number from the start to the end of their study, but a statistical relationship with age per se was not evaluated.

The ophthalmoscopic findings of VHL disease are divided into two main groups: angiomatous versus non-angiomatous lesions.

##  Angiomatous Tumors or Retinal Capillary Hemangiomas

**Figure 1:**
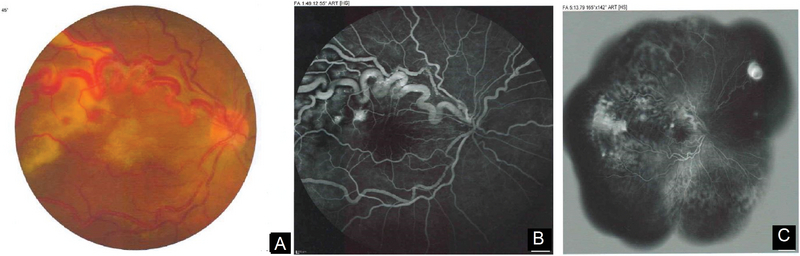
(A) Color fundus photograph of an exudative extrapapillary RCH. Note enlarged and tortuous feeding vessels passing toward a peripheral RCH, in addition to small RCHs in macula. (B) Wide field fundus angiogram. (C) Montage modality of angiography for more peripheral lesions.

Similar to other retinal lesions, retinal capillary hemangiomas are characterized by location, type, and size of the tumor. These tumors can be classified on the basis of morphology (endophytic, exophytic, and sessile), location within the retina (peripheral and juxtapapillary), and effects on the retina (exudative form and tractional form).^[40]^ The retinal capillary hemangioma is usually a well-circumscribed, round bulging with a red to orange color, with a variable appearance depending upon whether the tumor is endophytic, sessile, or exophytic. Blood vessels, as draining and feeding vessels, appear as the tumor grows larger and begins to be increasingly enlarged and tortuous [Figure 1(A)]. At this stage, extrapapillary RHs become exudative, with hard exudates and retinal edema near the tumor and/or in the macula. RHs at or near the optic disc have a distinct clinical appearance, making them difficult to discern with ophthalmoscopy when small in size or sessile in form. A localized fullness of the disc margin may be the primary finding, but with further growth, a bordered pink thickening becomes visible, with associated fine vessels in some cases. Feeder and draining vessels are typically not visible in juxtapapillary lesions. Although papillary hemangiomas sometimes show minimal growth over years, they usually lead to exudation eventually.

In a study by Wong et al it was reported that 85% of the affected eyes, manifested RHs exclusively in the peripheral retina (extrapapillary), 8% had RHs exclusively near the optic disc, and 7% had RHs in both the peripheral retina and juxtapapillary regions. Among the 421 eyes with peripheral RHs, the mean tumor count was 2.5. A subtle red or grayish spot with a few hundred micrometers of diameter was the initial clinical appearance of an extrapapillary RH, with an ophthalmoscopic appearance mimicking a dilated capillary, microaneurysm, or small intraretinal hemorrhage. Small tumors were sessile, but with growth, they often became more nodular. Earlier, Singh et al^[41]^ reported 174 retinal capillary hemangiomas in 86 eyes of 68 patients with VHL disease, where 83% were extrapapillary and 17% were juxtapapillary; 58% of RHs were 1.5 mm or smaller in size. In addition to data from distribution of RHs, the authors reported that juxtapapillary hemangiomas are frequently located at the temporal border of the optic disc.^[40,41]^

The natural course of a single capillary hemangioma in the retina can be of progression, stability, or regression. A majority of RHs grow over time, but occasionally, untreated tumors may remain static for a long period, and rarely they may regress spontaneously.^[45]^ Secondary effects such as exudation of subretinal or intraretinal spaces are often limited to the territory of the hemangioma but can be far enough to produce a macular star exudate. Retinal or vitreous hemorrhages are rarely observed, occurring in less than 3% of cases.^[46]^ Without treatment, large or multiple adjacent RHs may grow to displace the retinal structures and cause an exudative retinal detachment.

Fibrosis of the epiretinal space followed by posterior hyaloid contraction may accompany large RHs, causing macular epiretinal membrane with macular thickening, vitreomacular traction, or traction retinal detachment. As a rare complication, neovascularization of the iris can occur, which may lead to the development of neovascular glaucoma (NVG) and phthisis bulbi in eyes with multiple tumors. In a study involving a large cohort of patients, only 2% of eyes had neovascularization of the iris.^[43]^ Eventually, such eyes became phthisical or painful, requiring enucleation.

Visual function of patients with retinal capillary hemangiomas has been evaluated in some studies. Chew, in her prospective case series,^[35]^ reported that in 406 patients with VHL disease who had ocular involvement, visual acuity was 20/20 or better in 84.5% with hemangioblastomas, 3% were legally blind, and eventually, 8.2% had unilateral enucleations. In the cohort of the National Eye Institute, about 77% of eyes had a vision of 20/20 or better, and the prevalence of legal blindness, that is, vision less than 20/160 in the better-seeing eye, was 6%. However, to justify the burden of the disease, it was reported that approximately 20% of all patients with ocular VHL disease had at least some degree of unilateral visual impairment.

A longitudinal analysis with the purpose of characterization of visual function and ocular VHL progression was conducted in 2012.^[47]^ Two hundred and forty-nine participants of the study were followed-up for more than two years. Visual acuity, ocular manifestation of VHL disease, germline mutation in the VHL gene, demographic data, and patient characteristics were recorded in that study. The anatomic and functional ocular status was stable in most of the patients over a mean follow-up period of 8.2 years. In addition, 88% of eyes with ocular VHL disease at baseline, did not demonstrate RHs in a new retinal location, 70% remained stable in terms of RH number, and 79% revealed no change regarding the extent of RH involvement. In all 498 studied eyes, visual acuity was decreased by 5.1 letters across follow-up, with 16.1% decreasing by more than 10 letters.

In affected eyes, greater vision loss was related to the increase in RH number, the presence of juxtapapillary RHs, and formation of new RHs. There were correlations between younger age at onset of ocular VHL disease, bilateral ocular VHL disease, and missense or protein-truncating germline mutations and increase of anatomic involvement and functional worsening. Various stages of retinal involvement in retinal hemangiomas have been determined by some authors. Sigelman classified them into five stages. Stage one represents small hemangioma without any feeder vessels. In stage two, they appear as a nodule with prominence of only the draining vein. In stage three, both feeding artery and draining vein are present with or without retinal exudates. Partial and total exudative retinal detachment are observed in stages four and five, respectively.^[48]^ Similarly, Vail's classification^[49]^ includes the following stages:

Stage I: Angioma formation with feeding artery and draining vein

Stage II: Development of hemorrhages and exudation

Stage III: Massive exudation and retinal detachment

Stage IV: Uveitis, absolute glaucoma, and loss of the eye

The ophthalmoscopic findings of RHs are characteristic; thus, a good funduscopic examination is often sufficient to make an ocular VHL disease diagnosis. As mentioned earlier, small lesions may be difficult to be distinguished from microvascular abnormalities when other conditions are considered. On the other hand, some lesions may closely resemble larger RHs and may be difficult to be differentiated in some cases. Vasoproliferative tumor of the ocular fundus is the main differential diagnosis of large RHs.^[50]^ In addition, RHs in the presence of macular exudation can be misdiagnosed as retinal macroaneurysm or Coats' disease, and in some conditions such as vitreous hemorrhage which masks the tumor, dilated feeder vessels can be misdiagnosed as congenital retinal arteriovenous malformations and anastomoses between vessels of choroidal melanoma or retinoblastoma and retinal vessels. Unilateral papilledema and papillitis, juxtapapillary choroiditis, choroidal hemangioma, choroidal neovascularization, and amelanotic choroidal melanoma can mimic juxtapapillary retinal capillary hemangioma.^[51]^

##  Non-angiomatous Findings in Ocular VHL Disease

Some non-angiomatous retinal lesions associated with ocular VHL disease have been described. These lesions include “twin vessels,” “vascular hamartomas,” and “vascularized glial veils.” Schmidt, in 1995, found some unusual retinal lesions in his cases and called them vascular hamartomas.^[52]^ The lesions were described as small, flat, moss fiber-like, vascular lesions without enlarged afferent and efferent vessels. They were located in the superficial retina. Juxtapapillary fibrovascular membranes termed vascularized glial veils had been introduced by the same author seven years earlier. In the same year, de Jong et al defined twin vessels as a paired retinal arteriole and venule that were separated by less than the diameter of one venule and that extended for a distance of more than one disc diameter.^[53]^

In 2008, Wai et al reported a form of vascular proliferation as fine superficial vessels in 16 eyes of 14 patients, often found in juxtapapillary locations.^[54]^ These lesions most closely resembled vascularized glial veils. So that, the authors preferred the term “retinal vascular proliferation” to “vascularized glial veils” to include lesions that do not have a prominent fibrovascular component. Although the lesion was stable in 7 of 13 eyes, in the remaining cases, the lesion was so progressed that it led to vision loss.

##  Ancillary Tests

Fundus photography with wide-field modalities and montage methods seems to be an effective technique to monitor progression and growth of retinal hemangiomas, in addition to its use in detecting medium to large lesions [Figures 1(B) and 1(C)]. Ancillary testing becomes crucial in the evaluation of those patients with smaller lesions and those with peripheral exophytic hemangiomas to confirm the diagnosis. Ancillary tests for retinal hemangiomas may include fluorescein angiography (FA), indocyanine green angiography (ICG), optical coherence tomography (OCT), and ultrasonography.

Vascular dye tests such as FA and ICG angiography are advantageous diagnostic tools used in a range of ocular vascular growths such as retinal capillary hemangiomas, where FA is known as the most useful diagnostic tool because of the vascular nature of the tumor.^[55]^ FA and ICG angiography are critical in the assessment of any vascular lesion, helping in outlining the distinctive appearance, in addition to confirming any associated leakage.^[56]^

##  Fluorescein Angiography

Primary hyperfluorescence of the feeder artery during the arterial phase associated with fine capillary filling of the retinal lump and cumulative hyperfluorescence within the entire tumor during later phases is the main finding of retinal angiography in RHs.^[57]^ In more exophytic forms, FA may help clinicians to delineate the lesion, as well as the demarcation of the tortuous arterioles and venules. In circumstances that necessitate treatment, angiographic studies are also supportive in differentiating draining venules from feeding arterioles.^[58]^ In juxtapapillary hemangioblastoma, fine vascular configuration can be revealed on angiograms. Wide-angle angiography may be more beneficial, especially in finding peripheral lesions, which are more vulnerable to be missed because of their outlying position and subtle presentation. Although it is helpful in diagnosis, handling, and assessing the therapy, it is not required to perform FA on all cases of characteristic capillary hemangioma.

##  Indocyanine Green Angiography

ICG may also be valuable in the assessment of RH, although, generally, it is used to assess choroidal vasculature. Filling of the angiomas creates early, demarcated hyperfluorescence on ICG that is in communication with retinal vasculature. Absence of choroidal contribution on ICG angiography supports the diagnosis of RH. ICG is useful in distinguishing clinically similar situations, such as focalized choroidal hemangioma, which would be limited to the choroidal level.^[40]^ Associated retinal hemorrhages or retinal vascular leakage may be discovered by the blockage of adjacent vasculature on ICG, as an adjunct to FA. Indocyanine green-mediated photothrombosis has also been reported for the treatment of RHs.^[59]^

##  Ultrasonography

Ultrasound is a critical modality in assessing intraocular tumors. Evaluating size, diameter, and internal echogenicity, B-scan remains an important test in approach to ocular tumors. In RHs, B-scan shows a well-demarcated retinal lesion without choroidal properties. Ultrasonography is predominantly helpful in the presence of opaque media.^[60]^ Moreover, an RH is characterized on A-scan by an opening spike with secondary high internal reflectivity.^[40]^

##  Other Diagnostic Tests

Sequence investigation of the VHL gene, can be important in the evaluation of RHs, as these assist in the diagnosis of VHL disease. OCT may be used to discover macular edema and epiretinal membranes, as well as to evaluate features of a juxtapapillary RH.

##  Structural and Molecular Pathology Findings

RHs have two main components: glial proliferation and endothelial or vascular proliferation. The primary event is controversial. In fact, it is not determined whether the primary glial proliferation causes secondary vascular proliferation or glial proliferation follows endothelial proliferation.

Primary findings were of those studies conducted nearly half a century ago.^[61,62]^ It was shown that proliferation of glial cells surrounded the proliferated endothelial cells, which were arranged in well-formed vessels or small nests. The most prominent feature was a collection of large capillary-sized blood vessels that substituted the full thickness sensory retina. In addition, primary ultrastructural studies indicated that both endothelial and perithelial elements of the large capillary unit were morphologically regular. Thus, capillary hemangioma was a more exact histopathologic description for the von Hippel angioma than hemangioblastoma or hemangioendothelioma. This structure was reinforced in a study with the purpose of immunohistological evaluation of RH in two cases, where the authors reported that ultrastructural findings in both eyes contained endothelial/pericyte-lined vascular networks, shortened stromal cells, and fat and vacuolated stromal cells with ultrastructural features consistent with glial cells.^[63]^ Pathological findings of extrapapillary hemangiomas treated with xenon photocoagulation, argon laser therapy, and cryotherapy showed occlusion of the vascular channels, fibrous metaplasia of the retinal pigment epithelium, and secondary gliosis.^[64]^

Chan et al in 2007 tried to summarize discoveries in VHL pathology.^[65]^ They concluded that loss of heterozygosity (LOH) within the VHL gene is noticed in the stromal cells which originate from vascular/endothelial ancestry and form a unit with glial cells in retinal hemangiomas. Parallel LOH has been established in VHL disease-related CNS hemangioblastomas, which are histopathologically analogous to RH. This finding is not compatible with the old belief about stromal cells, where they were supposed to be lipidized astrocytes or glial cells.

Chan et al have suggested that increase of hypoxia-inducible factor (HIF), VEGF, and ubiquitin are found in ocular hemangioblastomas. In their study, tumorlet cells were introduced as small, poorly differentiated cells with dense nuclei and minor cytoplasm, owning several stem cell and immunologic markers such as CD133 65. Later in 2015, they found their pathological results to be consistent with previously described structure for RHs.^[66]^

The mainstream of evidence supports the understanding that retinal hemangioma is a vascular growth arising as a malformed vascular unit containing an arteriole, capillaries, and venule with consequent growing of all its components. Endothelial lineage of stromal cells and peripherally located glial cells indicates endothelial growth as the primary event. More discoveries about the molecular happenings in RH corpus may provide a therapeutic target for retinal hemangiomas.

##  Ocular VHL Disease and Pregnancy 

In 2009, Hayden et al published a report of spinal hemangioma worsening in a pregnant VHL patient.^[67]^ They believed that hormonal alterations during gestation hastened the growth of hemangioblastomas, leading to new symptoms. Following the report, Frantzen et al in their retrospective study involving 29 patients^[68]^ claimed that pregnancy causes progression of cerebellar hemangioblastomas. However, in 2015, Binderup et al criticized the two previous studies for their retrospective nature, short follow-up period, and limited samples.^[69]^ Their cohort study exclusively compared hemangioblastoma risk in pregnancy with age-matched non-pregnant years in the same female group and evaluated the influence of pregnancy on both CNS and RH progression. In contrast to the initial impression, they reported that gestation was associated with even lower rates of new tumor formation compared with the non-pregnancy period. It seems that pregnancy does not have any effect on the growth of preexisting RHs or development of new lesions.^[69]^

##  Treatment of Ocular VHL Disease

Early diagnosis, making decision for treatment versus observation monitoring for further growth after primary treatment, management of secondary retinal complications and treatment complications are some of the challenges in the field of ocular VHL disease management. The main goal of therapy is destruction of the lesion in order to reduce secondary damage to the retina. Apparently, small tumors in the early phases of growth can be destroyed quite readily with minimal risk associated with the treatment. In contrast, as hemangioblastomas grow, it will be more difficult to ablate them, with higher risk of damage induced by the therapeutic procedures. Accordingly, it seems that the principal issue in the management of ocular VHL disease is identification of RHs in the early stages of development, as well as timely therapy. However, even effectively managed, RHs can cause life-long visual loss in up to 25% of cases.^[46]^

In order to achieve suitable management of RHs, an optimal approach should be initiated with adequate surveillance. Indirect ophthalmoscopic and biomicroscopic examination of the retina, with occasional usage of ancillary tests are indicated every year for individuals with VHL disease, beginning in early childhood. Documentation of any ocular lesions allows the clinician to select one of the treatment modalities, including observation,^[70]^ laser photocoagulation,^[71,72,73,74]^ cryotherapy,^[75,76,77,78]^ plaque radiotherapy,^[79]^ vitreoretinal surgery,^[80,81]^ external beam radiation, proton beam radiation, photodynamic therapy (PDT),^[82,83]^ trans-pupillary thermotherapy, intraocular injection of anti-VEGF drugs or triamcinolone acetonide (TA).

Relative regression signs in a small RH may be detected with close surveillance. In a study by Singh et al in 2002,^[41]^ regarding management techniques in 68 patients who had been treated between 1974 and 1999, 82% of the 77 RHs that were primarily observed persisted stable for a median follow-up of 7 years. Most of the RHs that were initially observed were 3.0 mm or smaller in size and almost the same percentage of juxtapapillary and extrapapillary RH were present. The authors reported that for the extrapapillary RH, efficiency of observation as a way of management was higher for hemangiomas that were 1.5 mm or less in size than for larger RH. In their opinion, cautious observation is indicated in a reliable case if the RH is very small (up to 500 μm), is not associated with exudation, and is not vision threatening because of a nasal locality.

##  Ablative Treatment of Extrapapillary RHs

Ablative treatment includes various modalities such as thermal laser photocoagulation, cryotherapy, radiation (including brachytherapy, external beam radiation, and proton beam radiation), PDT, and trans-pupillary thermotherapy.

##  Laser Photocoagulation

Laser photocoagulation may be applied in numerous sessions and is most effective in lesions that are 1.5 mm or smaller,^[41]^ but it also has been used for RHs up to 4.5 mm with real regression.^[72]^ Although the modality is classically used for peripheral tumors, the effectiveness of laser photocoagulation for juxtapapillary RHs has been presented in some studies. Permanent scotoma, poor visual outcome due to juxtaposition to optic nerve and posterior position are possible complications of laser photocoagulation for optic nerve hemangiomas.^[70]^ Various laser types, including argon, yellow dye, diode, green, and krypton, have all been used.^[41]^ Efficacy of all kinds of laser photocoagulation has been confirmed in several studies. For example, in Singh's study, argon/diode laser photocoagulation, applied over a mean of 1.2 sessions was 100% effective in treating retinal hemangiomas that were smaller than 1.5 mm. Vascular lesions such as RHs can absorb yellow laser more than other laser wavelengths based on the absorption range of oxyhemoglobin;^[84]^ therefore, green and yellow wavelengths are usually used, with longer burn intervals (0.2–0.4 seconds) than would be usual for panretinal photocoagulation or laser retinopexy, and with power adequate to generate whitening in the zone of the burn. No randomized trial for ablative laser type or technique has been performed.

There are different types of photocoagulation techniques including direct photocoagulation of the RH,^[64,70,71]^ treatment of the feeder vessel,^[73]^ or combination of both the routes. Because of the possibility of bleeding caused by laser treatment, in some studies, blood vessel photocoagulation has been recommended to diminish blood flow of the retinal lumps.^[85]^ Blodi's study^[73]^ revealed that both direct and feeder vessel photocoagulation procedures are effective, but laser photocoagulation of the feeder arterioles may cause the need for further laser sessions. In cases with small tumors, burns are generally restricted to an area appropriate to blanch the entire lesion surface; feeder vessels in these tumors are so small, if detectable at all. An effective laser photocoagulation yields a chorioretinal scar, which is occasionally associated with a wasted, pale pink remnant of the lesion, and other times with complete vanishing of the RH. A visible regressed lesion may be a sign of destruction sufficient to prevent further growth or exudation, but cured areas must be followed over time for any signal of a still viable RH. If required, retreatment technique will be the same. In larger tumors, it will be challenging to apply intense photocoagulation throughout the depth of the tumor. Long duration burns (often more than 0.4 sec) with a lower power setting may be involved in the treatment of masses with a size between 1.5 and 4.0 mm in order to cause progressive whitening over the path of the burn. Although the visibility of the feeder vessels is enhanced in large tumors, photocoagulation of the feeding arterioles has not considerably increased the probabilities of success or lowered the risks associated with the procedure.

Occasionally, indirect laser photocoagulation is applied for tumors that are present anterior to the equator, where slit lamp laser delivery may be difficult. In addition, reasonable accomplishment rates have been reported with the use of laser endophotocoagulation as an adjunct to vitreoretinal surgery^[86]^.

Following laser therapy of both small and large tumors, appearance of scarce intraretinal or preretinal hemorrhage on the treated tumor is common, but vitreous hemorrhage is rare. Other reported complications of laser photocoagulation are subretinal fluid accumulation and exudative retinal detachment.^[87]^

It is notable that for laser therapy, sessile masses are more suitable than very nodular ones, and exudation, preexisting hemorrhage, and epiretinal fibrosis may have a negative effect on proper laser treatment. In addition, practicability and usefulness of therapy depends on a number of issues such as tumor location, grade of exudation, presence of retinal detachment, associated chorioretinal scarring, location relative to the position of any previous scleral buckling, the number and appearance of other viable tumors, concomitant retinal vascular alterations or vascular proliferation, and reaction to prior treatment(s).

McCabe et al reported a large series of patients that had been treated with laser photocoagulation with variable functional results. They concluded that due to the absence of standardization of laser photocoagulation, the functional consequences were not comparable.^[88]^ Huang et al reported the individual tumoral response in a total of 39 retinal capillary hemangiomas using a 532-nm laser system^[87]^. RHs < 1 optic disc diameter were directly photocoagulated. For RHs > 1 disc diameter, the nourishing vessel was photocoagulated first, followed by multiple tumor bulk photocoagulations until tumor atrophy occurred. Of all tumor bodies treated with photocoagulation, 82.4% were controlled at the last visit. The percentage of tumors treated with photocoagulation was 76.5%, which was similar to the rate (74%) reported by Singh et al.^[41,87]^

##  Cryotherapy

Although there are few reports about the efficiency of laser photocoagulation for huge RHs, according to the experience of the authors of this review, a substantial number of RHs in this size range are not destroyed even following several sessions of laser photocoagulation. Along with laser therapy, trans-scleral cryotherapy can be effective for the destruction of these masses, even in the presence of simultaneous exudation, hemorrhage, or fibrosis. Similarly, Singh et al reported that accompanied with laser photocoagulation, cryotherapy is the backbone of treatment for RHs > 1.5 mm in diameter and are placed anteriorly with subretinal fluid.^[41]^ For more anterior tumors, cryotherapy may be applied trans-conjunctivally in the office setting, while for posteriorly located tumors, a conjunctival incision may be needed to provide proper placement of the cryo probe. Numerous studies have shown cryotherapy effectiveness, particularly when RHs < 3.75 mm.^[64,71,76,77,78]^ As described by Welch, cryotherapy should be applied till the ice ball completely encloses the RH.^[72]^ Double freeze–thaw technique is usually used for cryotherapy. Use of cryotherapy seems to be associated with a more post-treatment exudative response than the use of laser photocoagulation.

##  Radiotherapy

External beam radiotherapy, proton beam radiotherapy,^[89]^ and plaque radiotherapy^[79]^ are additional modalities for large tumors (> 4.0 mm in diameter), which demonstrate poor response to cryotherapy and laser photocoagulation.

Although commonly used in the management of choroidal hemangioma, brachytherapy was not used for the treatment of RH until 1998.^[90]^ Kreusel and colleagues reported the use of ruthenium-106 brachytherapy for treatment of 25 eyes.^[79]^ The mean width of treated hemangiomas was 3.8 mm, the mean apex dose was 126 ± 36 Gy, and the mean scleral contact dose was 518 ± 85 Gy. Dose was transported over five to seven days. Finally, the authors reported destruction of 23 out of 25 masses with a single radiotherapy session. Nine eyes showed post-radiation complication including severe visual acuity reduction, a persisting exudative retinal detachment, or a recurrent traction detachment. Risk factors for these complications included pre-treatment exudative retinal detachment and tumor size > 3.75 mm. It is recommended to restrict the use of brachytherapy to moderately sized RHs < 3.75 mm without exudative retinal detachment. In Singh's series, a total of four extrapapillary RHs with a mean size of 4.5 mm (3–6 mm) were treated with iodine 125 plaque, delivering an average apical dose of 34.8 Gy.^[41]^

For the first time, Palmer and Gragoudas successfully treated one patient with a juxtapapillary hemangioma with proton beam therapy.^[89]^ Sixteen years later, the report of Seibel et al in 2014, described the treatment of a series of eight patients with symptomatic retinal papillary capillary hemangioma with proton beam therapy.^[91]^ This series of progressive stages of papillary hemangioma demonstrated an acceptable anatomic outcome after proton beam therapy. However, poor early visual acuity attributable to central exudation and long persisting macular edema compromised the final visual outcome. The authors advise that even in those patients ineffectively treated with laser photocoagulation or PDT, exudation may entirely resolve when proton beam therapy is used as a secondary treatment. Although proton beam therapy is a therapeutic option in the treatment of retinal papillary hemangioma, according to these findings, the treatment will remain challenging.

Not widely used, application of external beam radiation has been pronounced in advanced cases without favorable long-term consequence.^[92]^

##  Transpupillary Thermotherapy

Transpupillary thermotherapy (TTT) has an uncertain role in the treatment of RHs. There are limited experiences in the treatment of VHL with this modality. Parmar in 2000 and Singh in 2002 reported treatment of juxtapapillary RHs with trans-pupillary thermotherapy in one and three patients, respectively.^[93]^ In the first case, TTT caused an improvement in visual acuity from counting fingers to 6/24 and an obvious decline in exudates surrounding the hemangioma. However, in three patients in the second study, there was no apparent effect in two cases, although, in the third patient, it caused whole fibrosis of the juxtapapillary hemangioma associated with concomitant optic atrophy.

##  Photodynamic Therapy (PDT)

PDT is increasingly used for the treatment of RH, mainly for large tumors or juxtapapillary hemangiomas. Studies using verteporfin PDT for both juxtapapillary and peripheral RH have revealed diverse anatomical and functional conclusions.^[94]^ Schmidt-Erfurth et al, in a prospective non-comparative case series, reported that PDT is effective in reducing tumor dimension and exudative activity of optic disc head hemangiomas.^[82]^ A study reported the effect of PDT on four eyes with juxtapapillary RH. Two eyes was treated by full PDF while two other eyes was treated by half PDT. Tumor regression was observed in two eyes while no change was remarked in tumor size in other eyes.^[94]^ The study showed that PDT can be effective in decreasing macular edema associated with RH, but this does not constantly correspond with an enhancement of visual acuity, particularly for juxtapapillary tumors. In a retrospective analysis of all patients with RH treated with PDT between 2003 and 2010,^[95]^ it was concluded that the response of RHs to PDT is unpredictable; however, PDT may be used in juxtapapillary tumors where radiotherapy or cryotherapy is expected to result in simultaneous visual loss. Recently, in Huang's study,^[87]^ it was shown that good long-term vision outcomes were acquired in some peripheral RH cases after treatment; worsening of vision was detected in other cases even when the lesion regressed or was stable, and complications such as macular edema and exudates were resolved. In the latter study, it is suggested that PDT should be recommended for RH if available, but it is clear that therapies that are more effective are required for difficult RH cases, including juxtapapillary capillary hemangiomas and those with exudative retinal detachment or macular edema. In addition, the authors of this review have not found PDT to be effective enough to be used routinely.

##  Surgical Excision of Extrapapillary RHs 

As mentioned before, there are significant risks and low success rates of ablation in very large RHs. Because of that, surgical excision of RHs during vitrectomy is sometimes employed. In addition, vitreoretinal intervention is frequently essential for larger RHs complicated by rhegmatogenous or tractional retinal detachment.^[41]^ In a retrospective case series of three patients,^[96]^ tumors 7 mm to 9 mm in diameter were removed via internal en bloc surgical resection using a bimanual technique. According to patients' favorable outcomes, the authors suggested that surgical resection is a choice for those with large RHs. Gaudric and colleagues reported a case series of 23 eyes that underwent vitreoretinal surgery for progressive ocular VHL disease, in which 14 eyes received cryotherapy or laser endophotocoagulation as adjunct to vitrectomy and the other 9 eyes had surgical removal of RHs.^[86]^ For the nine eyes undergoing RH excision, a mean of two operations was needed, and eight out of nine eyes had an attached retina six months following the surgery. However, NVG and new tumor development occurred in four eyes between four and eight years after the RH excision, and visual acuity in remaining eyes was poor (20/320 or worse). The authors of the study claimed that vitreoretinal surgery is an effective treatment for severe VHL retinal hemangiomas, as in most cases, surgery enhanced or extended visual function. However, we believe that the combination of high rate of RH recurrence and post-surgery proliferative vitreoretinopathy restricts the accomplishment of this approach.

##  New Concepts in Ocular VHL Disease Treatment

##  Anti-VEGF therapy

Observation of the extremely vascularized nature of lesions in VHL disease led to a hypothesis that VEGF, a HIF-inducible protein and potent mediator of angiogenesis and vascular permeability, might be central in RHs progression, and there are numerous lines of evidence fingering VEGF expression in the pathogenesis of VHL disease. VEGF protein levels are raised in specimens from renal carcinomas demonstrating mutations of VHL.^[97,98,99]^

Anti-VEGFs decrease the amount of intraretinal edema and bleed, thereby reducing the size of the lesion and the nourishing vessels. Anti-VEGFs have been displayed to accelerate the clearance of hemorrhages and exudation, which leads to the improvement of visual acuity along with diminished probabilities of re-bleeding.^[100]^ In a retrospective interventional case series,^[101]^ it was shown that intravitreal anti-VEGF agents, unaided or in combination with other treatment modalities, may recover visual acuity, but additional trials assessing the dose, the quantity of injections, and the route of administration will be essential in evolving antiangiogenic therapies for RH. Earlier, in one prospective study, Dahr and coworkers assessed intravitreous pegaptanib sodium (3 mg) in five patients manifesting juxtapapillary or extrapapillary RH.^[102]^ Pegaptanib sodium was administered every six weeks for at least six injections, and two of five patients finished the course and one year of follow-up. These two patients experienced a reduction in exudation, but no alteration in dimensions of the tumors. The other three patients demonstrated advancement of ocular disease and did not finish the course of treatment. In another interventional case series, Wong et al evaluated the effect of intravitreal ranibizumab in five patients with retinal hemangiomas not responsive to standard treatments.^[103]^ Ranibizumab (0.5 mg) was administrated every one month for six months, with supplementary treatment through 12 months. Participants received an average of 10 injections over a mean of 47 weeks. Visual acuity declined by nine letters and there was no consistent reduction in RH tumor size or improvement in exudation.

Recently, Agarwal et al^[100]^ reported a case with RH located in the perifoveal region treated with two monthly intravitreal injections of bevacizumab followed by laser photocoagulation of feeder arterioles. This combination therapy resulted in a resolution of exudation, bleeding, and macular edema with improvement in visual acuity.

##  Intravitreal Propranolol

The therapeutic effect of intravitreal propranolol on retinal capillary hemangioma was reported in a patient with VHL. No short-term adverse effects except a mild transient inflammatory response were observed in this case report.^[104]^ Fluorescein leakage was decreased from the RHs located on the optic nerve head and in the inferonasal retinal periphery one month after the second intravitreal injection of propranolol. Decrease of the hemangioma vascularity and augmentation of its fibrosis associated with the attenuation of the feeder vessel were observed. Electroretinogram done one month after the first injection revealed no retinal toxicity.

##  Treatment of Juxtapapillary RHs

Reduction of visual acuity, visual field loss, and development of central scotomas have limited the ablative therapies in managing juxtapapillary hemangiomas. According to findings of multiple studies mentioned earlier, reduced visual function has been associated with thermal laser photocoagulation.^[105]^ On the other hand, PDT with verteporfin, which seems to have a safer profile than laser photocoagulation, has demonstrated incomplete success and fairly varied results.^[82,106]^ In a retrospective case series in 2000,^[105]^ it was claimed that close follow-up and multiple treatments with argon laser are probably the best therapeutic approach, as if left untreated, papillary angiomatous lesions may evolve to exudative retinal detachment with severe visual acuity decreases. Given these considerations, asymptomatic juxtapapillary RHs are usually observed with the hope that some lesions remain relatively static for long periods. It is believed that exudation may wax and wane and can remain compatible with good vision unless the central macula becomes chronically affected. In the absence of safe ablative choices, treatment is typically limited to alleviating the exudation affecting vision. Pharmacotherapy using corticosteroids or VEGF antagonists, proton beam therapy, and PDT may decrease exudation in some patients, but risks of therapy must be carefully evaluated, and success is often limited.^[91,102,103]^

##  Treatment of Retinal Vascular Proliferation

As mentioned earlier in the clinical manifestations section, a series of VHL patients have been described with an infrequent retinal proliferation varying in natural history, phenotype, and treatment.^[54]^ They do not appear to carry the equal risk of vitreous hemorrhage or traction retinal detachment as neovascularization in other ischemic retinopathies, and they sometimes regress spontaneously. When they grow large to be more contractile due to fibrosis adjacent to the fovea, they can reduce visual acuity. It is essential to distinguish these lesions from RHs. Retinal vascular proliferation does not typically cause exudation and does not regress easily in response to laser photocoagulation, in part due to its epiretinal situation. In cases of recognized progression, these lesions may be effectively addressed by excision with vitrectomy and membrane peeling.^[54]^

##  SUMMARY

RH is a benign vascular tumor of the neurosensory retina or optic disc that sometimes appears as a sporadic lesion, but classically manifests as one or more tumors in the setting of VHL disease. Referral of a patient with RH for clinical and genetic testing for VHL disease may be lifesaving, given the deadly nature of manifestations such as CNS hemangioblastoma and RCC, and given the profits of surveillance and early treatment in affected individuals. Other essential constituents of ophthalmologic management include suitable surveillance of patients with VHL disease for growth or development of RHs and retinal vascular proliferation. RHs developing in proximity to the optic disc exhibit a specific challenge, because they are normally not safe to abolish using traditional ablative procedures. Improved knowledge of the molecular pathology of VHL disease presents possibility for development of non-ablative therapies to stop growth or accelerate the regression of RH in this condition.

##  Financial Support and Sponsorship

Nil.

##  Conflicts of Interest

There are no conflicts of interest.
